# 

**Published:** 1891-01

**Authors:** 


### Society Meetings.

### SEVENTH INTERNATIONAL CONGRESS OF HYGIENE AND DEMOGRAPHY.

*Editors Buffalo Medical and Surgical Journal:*

I am requested by the Honorary Secretaries of the Committee of Organization of the Seventh International Congress of Hygiene and Demography, to call attention to the fact that this Congress will be held in London during the week beginning August 10, 1891.

The Governments of all countries and municipalities, and all public health authorities, universities, colleges, and societies occupied in the study of the sciences, more or less immediately connected with hygiene, are invited to coöperate and appoint delegates to represent them at the Congress. The Prince of Wales will preside.

A Committee of Organization has been formed, of which Sir Douglas Galton is chairman, and Professor W. H. Corfield and Mr. Shirley F. Murphy are honorary secretaries. An exhibition of articles of hygienic interest will be held in connection with the Congress. The last of these Congresses was held in Vienna in 1887, and was attended by over 2,000 persons, and it is expected that the London meeting will be one of great magnitude and importance.

Very respectfully,

JOHN S. BILLINGS, M. D.,

*Member of the International Permanent Committee.*

WASHINGTON, D. C., October 27, 1890.

### BOOKS AND PAMPHLETS RECEIVED.

Transactions of the American Dermatological Association at its Fourteenth Meeting, held at Richfield Springs, N. Y., September 2, 3, and 4, 1890. Official report of the proceedings. By George Thomas Jackson, Secretary. New York. 1890.

Modern Treatment of Headaches. By Allan McLane Hamilton, M. D. The Physician's Leisure Library. No. 6, second edition. Detroit: George S. Davis. 1890. Price, cloth, 50c.; paper, 25c.

The Teaching and History of Mathematics in the United States. By Florian Cajori, M. S., (University of Wisconsin), formerly Professor of Applied Mathematics in the Tulane University of Louisiana, now Professor of Physics in Colorado College. Bureau of Education, Circular of Information No. 3. 1890. 8vo; pp. 400; paper. Washington: Government Printing Office. 1890.

Report of the Surgeon-General of the Army to the Secretary of War, for the fiscal year ending June 30, 1890. Washington: Government Printing Office. 1890.

Annual Report of the Treasurer of the United States. 1890. Washington: Government Printing Office.

Report of the Proceedings of the Fifteenth Annual Meeting of the Alumnæ Association of the Woman's Medical College of Pennsylvania, March 14, 1890. Philadelphia: Penfold Brothers. 1890.

Report for the Year 1889-90, presented by the Board of Managers of the Observatory of Yale University to the President and Fellows.

Treatment of Catarrh. By J. J. Stephens, M. D., Clinton, Mo. Reprint: St. Louis *Courier of Medicine*, September, 1890.

Remarks on the Albuminate of Iron. By John A. Ouchterlony, A. M., M. D., Professor of the Principles and Practice of Medicine and Clinical Medicine, Medical Department University of Louisville Reprint: *American Practitioner and News*. 1890.

The Relation of Bacteria to Practical Surgery. The Address in Surgery delivered before the Medical Society of the State of Pennsylvania, June 4, 1890. By John B. Roberts, A. M., M. D., Professor of Surgery in the Woman's Medical College, and in the Philadelphia Polyclinic. Philadelphia: William J. Dornan. 1890.

Quinine as a Prophylactic or Preventive against Malarial Fevers. New York: C. F. Bockringer and Sœhne. 1890.

*University Medical Magazine Bulletin*, December, 1890, containing: I. Dosage, Method of Injection, and Symptoms Produced by Subcutaneous Injections of the Lymph, as stated in Koch's paper. II. Specific Directions Accompanying the Lymph. III. Clinical Results of the Treatment of Tuberculosis by Koch's Method. IV. Methods of Investigation to be Pursued at the University Hospital. V. Berlin Correspondence.

Rupture of an Ectopic-Sac in the Sixth Month of Pregnancy. Abdominal Section and Recovery. By Drs. James Moran and Thomas H. Manley. New York: E. P. Coby & Co. 1890.

Two Cases of Fractured Skull. Recovery in one; death from chloroform in the other. By Thomas H. Manley, M. D., Visiting Surgeon to the Harlem Hospital, New York. Reprint: *The Medical News*, October 4, 1890.

Early Operation for Hare-Lip, with the Report of a Case, Illustrations, etc. By Thomas H. Manley, A. M., M. D. Reprint: *The Medical Age*, September 25, 1890.

Ueber die Ergebnisse der Totalexstirpation des Carcinomatösen Uterus. Von Professor Dr. Leopold und Dr. Münchmeyer in Dresden. Separatabdruck aus der *Münchener Medic. Wochenschrift*, Nr. 46. München, 1890. Druck der Akademischen Buchdruckerei von F. Straub.

Some Recent Cases in Pelvic and Abdominal Surgery. By L. S. McMurtry, M. D., of Louisville, Ky., Gynecologist to Sts. Mary and Elizabeth Hospital; Fellow of the American Association of Obstetricians and Gynecologists; Fellow of the Edinburgh Obstetrical Society, etc. Read before the Kentucky State Medical Society, May, 1890.

Galvanism in the Treatment of Corneal Opacities. By L. A. W. Alleman, M. A., M. D. Reprinted from the Brooklyn *Medical Journal*, November and December, 1890.

Imperforate Auditory Canals. By Seth S. Bishop, M. D., Surgeon to the Illinois Charitable Eye and Ear Infirmary, Chicago. Read before the Tenth International Congress. Reprint: *Journal American Medical Association*.

The Abuse of a Great Charity. By George M. Gould, M. D., Ophthalmologist to the Philadelphia Hospital. Reprint: *The Medical News*, November 22, 1890.

Early Exploratory Incision as an Aid to the Diagnosis of some Surgical Diseases of the Abdominal Cavity. By Edwin Ricketts, M. D., Cincinnati, O., Professor of Abdominal Surgery and Gynecology in the Cincinnati Polyclinic; Member of the American and British Medical Associations, etc. Reprint: *Transactions Medical Society of Virginia.* 1890.

Further Notes on the Chigger (Septus Irritans). By Dr. H. M. Whelpley, F. R. M. S. Reprint: *Popular Science News*, July, 1890.

Treatment of Uterine Fibro-Myomata by Abdominal Hysterectomy. By J. C. Irish, M. D., Lowell, Mass. Reprint: Boston *Medical and Surgical Journal*, September 25, 1890.

The Treatment of the Morphine Disease. By J. B. Mattison, M.D., Brooklyn, N. Y. Reprint: *The Therapeutic Gazette*, September 15, 1890.

*The Naturalist's Leisure Hour and Monthly Bulletin*, December, 1890. A. E. Foot, 4116 Elm avenue, Philadelphia.

Thirty-fifth Annual Announcement of the Kentucky School of Medicine. Louisville, Ky. Session of 1891.

HEALTH BULLETINS.

State Board of Health of Tennessee, November 20, and December 20, 1890.

State Board of Health of Michigan, December 5, 1890.

State Board of Health of New York, October and November, 1890.

Report of Deaths and Contagious Diseases, Newport, R. I., November, 1890.

Abstract of Sanitary Reports United States Marine Hospital Service. Volume V., Numbers 48, 49, 50, 51, and 52. 1890.

NOTICE TO CONTRIBUTORS. — We are glad to receive contributions from every one who knows anything of interest to the profession. Articles designed for publication in the JOURNAL should be handed in before the first day of the month. The Editors are not responsible for the views or opinions of contributors. All communications should be addressed to

284 FRANKLIN STREET, BUFFALO, N. Y.

